# SPRY2 is a novel MET interactor that regulates metastatic potential and differentiation in rhabdomyosarcoma

**DOI:** 10.1038/s41419-018-0261-2

**Published:** 2018-02-14

**Authors:** Masum Saini, Aakanksha Verma, Sam J. Mathew

**Affiliations:** Laboratory of Developmental Genetics, Regional Centre for Biotechnology, NCR Biotech Science Cluster, 3rd Milestone, Faridabad-Gurgaon Expressway, Faridabad, Haryana 121001 India

## Abstract

Rhabdomyosarcoma (RMS) is a predominantly pediatric soft-tissue cancer where the tumor cells exhibit characteristics of the developing skeletal muscle, and the two most common sub-types are embryonal and alveolar RMS. Elevated activation of the receptor tyrosine kinase (RTK) MET is frequent in RMS and is thought to cause increased tumor metastasis and lack of differentiation. However, the reasons underlying dysregulated MET expression and activation in RMS are not well understood. Therefore, we explored the role of Sprouty 2 (SPRY2), a modulator of RTK signaling, in regulating MET. We identify SPRY2 as a novel MET interactor that colocalizes with and binds MET in both embryonal and alveolar RMS. We find that depletion of SPRY2 leads to MET degradation, resulting in reduced migratory and clonogenic potential, and induction of differentiation in both embryonal and alveolar RMS, outcomes that are identical to depletion of MET. Activation of the ERK/MAPK pathway, known to be crucial for regulating cell migration and whose inhibition is required for myogenic differentiation, was downregulated upon depletion of MET or SPRY2. This provides a direct connection to the decreased migration and induction of differentiation upon depletion of MET or SPRY2. Thus, these data indicate that SPRY2 interacts with MET and stabilizes it in order to maintain signaling downstream of MET, which keeps the ERK/MAPK pathway active, resulting in metastatic potential and inhibition of differentiation in RMS. Our results identify a novel mechanism by which MET signaling is stabilized in RMS, and is a potential target for therapeutic intervention in RMS.

## Introduction

Rhabdomyosarcoma (RMS) is the most common pediatric soft-tissue sarcoma, accounting for about 3% of childhood cancers^1^. It is a relatively rare (~4.5 cases per million children annually), but aggressive malignancy^2–4^. The most common variants are embryonal (ERMS; ~67%) and alveolar rhabdomyosarcoma (ARMS; ~30%), which exhibit distinct clinical and molecular features^5,6^. Histopathologically, ERMS tumors are characterized by zones of hypo and hyper-cellularity, whereas loose nests of rounded cells interspersed by fibro-vascular septa are characteristic of ARMS^7^. ARMS is highly aggressive, frequently characterized by the chromosomal translocations t(2;13) involving *PAX3-FKHR*, or t(1;13) involving *PAX7-FKHR* fusion. ERMS has a relatively more favorable prognosis, and is associated with loss of heterozygosity of 11p15.5, p53 pathway disruption and RAS activation^8^.

RMS tumors show morphological similarities to developing muscle cells and express muscle differentiation markers such as MyoD, myogenin, and myosin heavy chain (MHC)^4,9–12^. Thus, RMS tumor cells recapitulate the embryonic myogenic program, although unlike embryonic myogenesis where cells exit the proliferative cycle upon terminal differentiation, the tumor cells persist in an undifferentiated state. Despite their resemblance to myogenic cells, the cell type of origin in RMS is debated. RMS have been proposed to arise from skeletal muscle stem cells (satellite cells), de-differentiation of terminally differentiated myogenic cells, or mesenchymal stem cells committing to the skeletal muscle lineage^13–15^.

Another common thread between mammalian myogenesis and RMS tumors is the expression of a receptor tyrosine kinase (RTK)–MET, by the myogenic progenitors and RMS cells^16–19^. MET was identified as a fusion oncogene in osteosarcoma, and is known to control cell proliferation, survival, and migration, in response to binding by its ligand hepatocyte growth factor (HGF) during developmental morphogenesis and in multiple cancer types^20,21^. During mammalian development, MET expression in myogenic precursors is required for their migration to target organs such as limbs^16,17^. During adult regenerative myogenesis, MET activates and regulates satellite cell migration, and controls myocyte fusion^22–24^. Interestingly, MET is overexpressed, aberrantly activated, essential for metastasis and inhibition of differentiation in RMS, and is a potential candidate for therapeutic targeting^18,19,25–27^. Thus, identification of MET regulators will be critical to understanding RMS pathology, and attenuating MET signaling by targeting MET or its regulators, could serve as intervention points in RMS patients.

Regulation of RTK signaling cascades is essential for physiological homeostasis^28^. The Sprouty (SPRY) family of proteins are important modulators of RTK signaling and SPRY2, a member of the family, functions as a bimodal regulator^29,30^. Versatility of SPRY2 in modulating RTK-mediated signaling is cell type, and RTK context dependent, which can result in opposing effects, potentiating or dampening signals transduced from RTKs^30,31^. While SPRY2 inhibits fibroblast growth factor (FGF)-mediated extracellular-signal-regulated kinase (ERK) signaling by preventing RAF activation, it augments epidermal growth factor receptor (EGFR)-induced ERK signaling, by inhibiting EGFR endocytosis and degradation^32,33^. SPRY2 also exhibits contrasting tumor suppressive or oncogenic roles in different cancer contexts^34–36^. For example, overexpression of SPRY2 negatively regulated HGF-mediated ERK and AKT signaling in human leiomyosarcoma, whereas SPRY2 overexpression increased MET activation resulting in enhanced cell migration and invasion in colonic adenocarcinomas^35,36^.

Association of MET activity with enhanced metastatic potential and inhibition of differentiation underscores the importance of understanding MET regulation in RMS. Since regulation of MET in RMS is largely unexplored and reports indicate that SPRY2 can alter MET signaling in cancers, we carefully analyzed MET, SPRY2 and their role in RMS, using representative RMS cell lines. Loss of SPRY2 function led to a significant reduction in MET protein levels in RMS cells, mediated primarily by the proteasomal pathway in ERMS and lysosomal pathway in ARMS. We uncovered that MET and SPRY2 interact physically and colocalize with each other in RMS cells. Notably, knockdown of SPRY2 or MET lead to similar functional outcomes in RMS cells, mediated by reduced ERK activation resulting in decreased migratory potential, and inducing differentiation. Thus, our study shows that SPRY2 is a key interactor and regulator of MET in RMS, functioning to stabilize the MET receptor to sustain downstream signaling, which is essential for maintenance of migratory, metastatic and clonogenic capabilities, as well as to inhibit differentiation in RMS.

## Results

### MET and SPRY2 expression varies between RMS cell lines

**Fig. 1:**
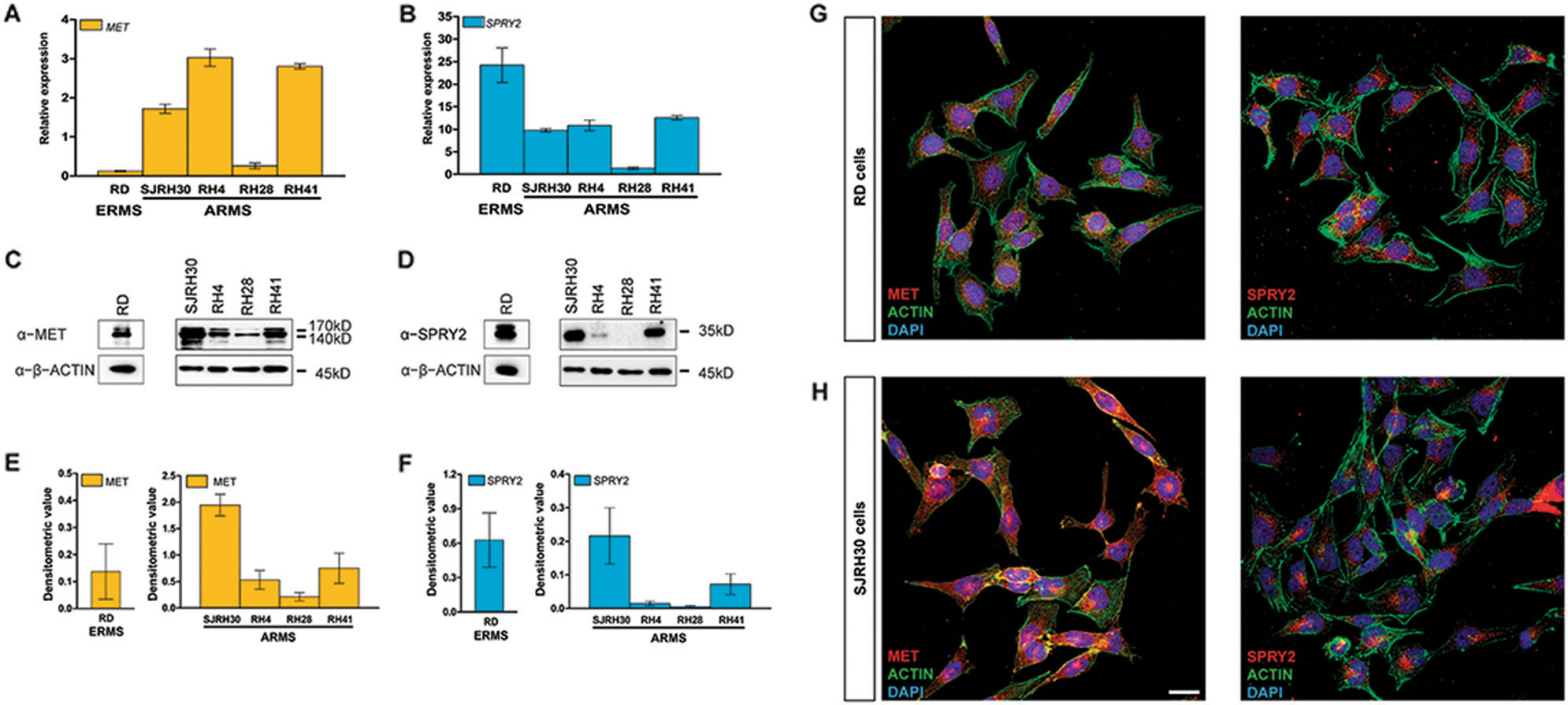
MET and SPRY2 expression varies across RMS cell lines. Quantification of *MET* (**a**) and *SPRY2* (**b**) transcripts in all five RMS cell lines was carried out by quantitative real-time PCR (qPCR). RD (ERMS) cells have lower levels of MET but higher levels of SPRY2 transcripts as compared to ARMS cell lines used in the study. *GAPDH* and *HPRT* were used for normalization. Representative immunoblots and densitometric analyses normalized to β-ACTIN show protein expression of MET (**c**, **e**) and SPRY2 (**d**,** f**) is variable in different RMS cell lines similar to the variability observed in their transcript levels (**a** and **b**, respectively). As with the transcript expression profiles, RD (ERMS) cells show lesser MET but higher SPRY2 protein expression compared to ARMS cell lines. Note that the *Y* axes of the densitometry graphs have variable scales owing to varying levels of expression. Immunofluorescence confocal imaging shows cytoplasmic localization of MET (red, left panels) and SPRY2 (red, right panels) in RD (**g**) and SJRH30 (**h**) cells, along with phalloidin labeling the actin fibers (green) and nuclear staining with DAPI (blue). Scale bar=25 µm

MET is upregulated in RMS^18,19^, and SPRY2 has been reported to regulate RTK signaling^32,33^. No reports exist on SPRY2 as a regulator of MET signaling in RMS. Therefore, to ascertain whether SPRY2 has any role in regulating MET in RMS, we characterized SPRY2 and MET expression in embryonal (RD, representative ERMS cell line) and alveolar (SJRH30, RH4, RH28, RH41) RMS cell lines. We found that MET transcript (Fig. 1a) and protein (Fig. 1c, e) expression was higher in ARMS compared to ERMS cells, whereas SPRY2 transcript (Fig. 1b) and protein (Fig. 1d, f) expression was higher in ERMS than ARMS cells. Expression of MET and SPRY2 proteins varied across the different RMS cell lines, but broadly in ARMS, cell lines with higher MET levels also exhibited elevated SPRY2 (Fig. 1c–f). MET and SPRY2 exhibited cytoplasmic localization in RD and SJRH30 cells (Fig. 1g, h). Among the ARMS cell lines, SJRH30 showed highest levels of MET and SPRY2 (Fig. 1e, f) and was chosen as the representative ARMS cell line, along with RD cells (ERMS), to investigate the regulation of MET signaling by SPRY2.

### SPRY2 regulates levels of MET receptor in RMS

**Fig. 2:**
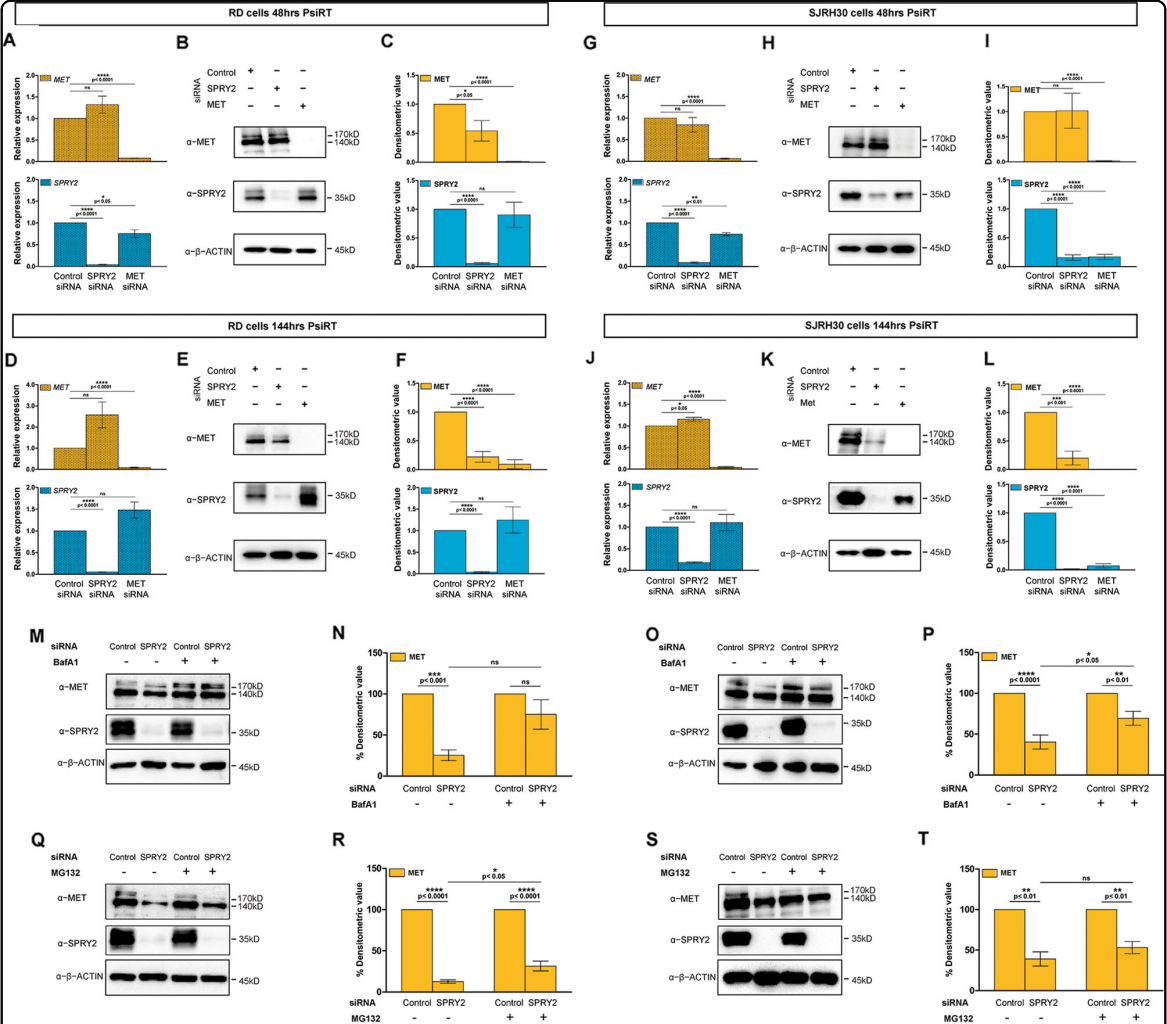
MET protein levels depend on *SPRY2* expression in ERMS and ARMS cells. Relative quantification of *MET* and *SPRY2* transcripts by qPCR at 48 and 144-h post MET, SPRY2, or control siRNA transfection in RD (**a**,** d**) and SJRH30 (**g**, **j**) RMS cells. The transcript levels were normalized to control genes, *GAPDH* and *HPRT*, and compared to control siRNA treated RMS cells. MET and SPRY2 siRNAs show >90% knockdown of their specific transcripts, upper and lower bar graphs respectively, in RD cells at 48-h (**a**) and 144-h (**d**) post transfection. MET siRNA shows similar knockdown efficiency of >90% in silencing MET transcripts (upper bar graphs) in SJRH30 cells at 48-h (**g**) and 144-h (**j**) post transfection. A knockdown of >90% and ~82% was observed in SPRY2 transcript levels (lower bar graphs) in SJRH30 cells at 48-h (**g**) and 144-h (**j**) post transfection respectively. Representative western blots of MET or SPRY2 siRNA transfected RD (**b**,** e**) and SJRH30 (**h**, **k**) cell lysates show efficient knockdown of MET (upper panels) and SPRY2 (middle panels), as compared to control siRNA transfected cells, at 48-h (**b**, **h**) that is maintained at 144-h (**e**,** k**) post-transfection in both the cell lines. Densitometry shows significant decrease in MET protein levels in SPRY2 siRNA transfected RD cells at 48-h (**c**) and 144-h (**f**) post transfection, whereas the SPRY2 protein expression is unchanged in MET siRNA transfected RD cells at both the time points. MET levels are also significantly downregulated in SPRY2 siRNA transfected SJRH30 cells, but only at 144-h (**l**) post transfection. Notably, unlike RD cells, SPRY2 expression is significantly reduced in MET siRNA transfected SJRH30 cells at both 48-h (**i**) and 144-h (**l**) post transfection. RD and SJRH30 cells transfected with SPRY2 or control siRNA for ~96-h were treated with Bafilomycin A1, MG132, or vehicle and cell lysates were analyzed for MET stabilization (**m** to **t**). Representative western blots show levels of MET and SPRY2 proteins in siRNA transfected and Bafilomycin A1, MG132 or vehicle treated RD (**m**,**q**) and SJRH30 (**o**,**s**) cell lysates. Densitometry was performed using β-ACTIN as the loading control. In SPRY2 silenced RD cells, MG132 treatment (**r**) stabilizes MET levels significantly but not upon treatment with Bafilomycin A1 (**n**). Conversely, in SJRH30 cells, MET levels upon SPRY2 siRNA treatment are stabilized significantly by Bafilomycin A1 (**p**) and are not significantly stabilized by MG132 (**t**). The graphical data represent the mean ± SEM of a minimum of three independent experiments

Multiple reports in different cancer types indicate that SPRY2 regulates MET levels^34–36^. The MET ligand HGF, and ERK signaling, a downstream effector of RTKs, are known to induce SPRY2 expression^35,37^. However, no studies have investigated MET, SPRY2 and their interactions in RMS. Since we observed elevated MET and SPRY2 levels, as well as similar cytoplasmic localization of both, we examined whether they exert regulatory effects on each other by knocking them down individually using small interfering RNA (siRNA) in RD and SJRH30 cells (Fig. 2). We validated the knockdown at the transcript (Fig. 2a, d, g, j) and protein (Fig. 2c, f, i, l) levels and observed >80% knockdown efficiency at early (48-h) and late (144-h) time points post-siRNA transfection (PsiRT).

Upon MET knockdown, SPRY2 transcript levels decreased significantly at 48-h PsiRT in RD and SJRH30 (Fig. 2a, g, lower graphs) cells, indicating that MET signaling regulates SPRY2 transcriptional activation, as reported in leiomyosarcoma^35^. However, this effect was not seen 144-h PsiRT, suggesting that compensatory signals restore SPRY2 transcriptional activation (Fig. 2d, j, lower graphs). At the protein level upon MET knockdown, SPRY2 levels were unchanged in RD cells (Fig. 2b, e, middle panels and C, F, lower graphs), whereas it was significantly downregulated in SJRH30 cells at 48 and 144-h (Fig. 2h, k, middle panels and I, L, lower graphs). This suggests intrinsic differences between ERMS and ARMS, and is likely due to enhanced MET expression in ARMS compared to ERMS^38^.

Upon SPRY2 knockdown, we did not observe any effects on MET transcript levels in RD (Fig. 2a, d, upper graphs), or SJRH30 cells at 48-h (Fig. 2g, upper graph), but a subtle upregulation was apparent at 144-h (Fig. 2j, upper graph). At the protein level, SPRY2 knockdown resulted in a striking reduction in MET by 144-h in RD and SJRH30 cells, indicating that SPRY2 depletion leads to MET degradation (Fig. 2e, f, k, l upper panels). These results were validated using a separate set of siRNAs targeting MET and SPRY2 (Supplementary Figure 1). Reduction in MET protein following SPRY2 depletion cannot be transcriptional, since MET transcript levels were not reduced upon SPRY2 knockdown (Fig. 2a, d, g, j upper graphs). Our findings suggest that MET partly regulates SPRY2 transcriptional activation in RMS, and SPRY2 protein levels in ARMS. On the other hand, SPRY2 has negligible effect on MET transcript levels, but is required to maintain MET protein levels, indicating that SPRY2 might be a crucial factor regulating MET in RMS.

Therefore, we tested whether MET undergoes degradation when SPRY2 is silenced in RMS cells. MET is degraded by proteasomal and lysosomal pathways^39^. Upon SPRY2 depletion, proteasomal inhibition with MG132 prevented MET degradation in RD cells (Fig. 2q, r), and although not statistically significant, a trend towards MET stabilization upon Bafilomycin A1-mediated lysosomal inhibition was observed (Fig. 2m, n). Conversely, MET levels stabilized significantly upon Bafilomycin A1 treatment in SPRY2 silenced SJRH30 cells (Fig. 2o, p), whereas MET stabilization upon proteasomal inhibition was insignificant in these cells (Fig. 2s, t). These findings indicate that SPRY2 regulates MET levels by preventing its degradation, through distinct pathways in the two RMS sub-types.

### SPRY2 colocalizes and interacts with MET in RMS

**Fig. 3:**
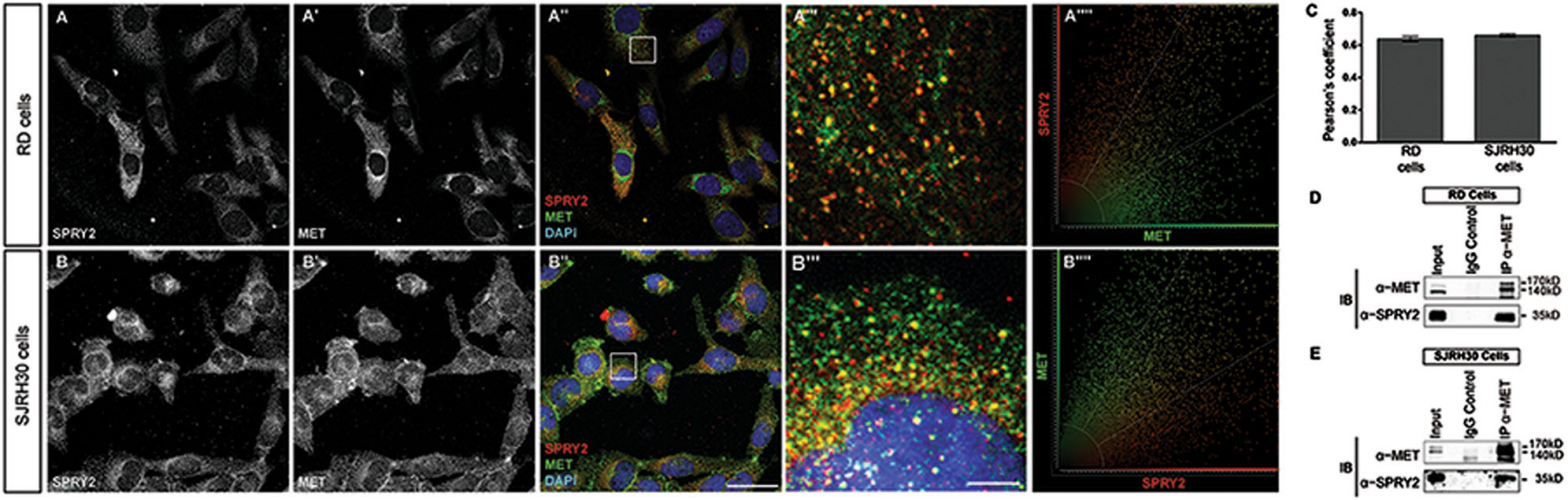
SPRY2 colocalizes and interacts with MET in RMS cells. Immunocytochemical staining shows that SPRY2 (red) and MET (green) colocalize with each other in RD (**a**–**a**″) and SJRH30 (**b**–**b**″) cells, suggesting that SPRY2 might interact with MET in embryonal and alveolar sub-types of RMS. Panels **a**′′′ and **b**′′′ represent magnified images (×10) of regions marked by white squares in **a**′′ and **b**′′. Nuclei are stained with DAPI (blue) and the scale bar is 50 µm and 5 µm in panels **b**″ and **b**′′′, respectively. Quantification of SPRY2-MET colocalization was performed as described in materials and methods. Representative scatter plots showing SPRY2 (red) and MET (green) colocalization signal in the entire cell in RD (**a**″″) and SJRH30 (**b**″″) cell lines. Pearson’s correlation co-efficient of the representative RD (**a**″″) and SJRH30 (**b**″″) scatter plots are 0.6608 and 0.6878, respectively. Bar graph showing the mean Pearson’s correlation coefficient for SPRY2-MET colocalization in RMS cells (**c**) (*n* = 17), where perfect correlation=1, no correlation=0 and error bar represents ± SEM. Equal concentrations of whole cell lysates from RD (**d**) and SJRH30 (**e**) cells were immunoprecipitated (IP) using anti-MET antibody. The immunocomplexes were probed with anti-MET and anti-SPRY2 antibodies by immunoblotting. IP was also performed using IgG isotype control. SPRY2 immunoprecipitated with MET in both RMS cell lines (**d**, **e**)

Since SPRY2 regulates MET and silencing SPRY2 decreased MET protein levels, we investigated whether SPRY2 interaction with MET is required for stabilizing and preventing MET degradation in RMS. To test this, we labeled MET and SPRY2 proteins by immunocytochemistry and both proteins localized to cytoplasmic punctae, colocalizing with each other as measured by Pearson’s correlation coefficient in RD and SJRH30 cells (Fig. 3a–a″″, b–b″″, c)^40^. To validate whether colocalization was an indication of biochemical interaction, we probed MET immunoprecipitates from RMS cell lysates with SPRY2 antibody. Interestingly, SPRY2 immunoprecipitated with MET in RD and SJRH30 cells (Fig. 3d, e). To our knowledge, no previous studies have shown that effects of SPRY2 on RTK regulation are mediated by a physical interaction between SPRY2 and the RTK. Thus, our findings indicate that SPRY2 and MET interact, which possibly is crucial to MET stability.

MET regulates cell proliferation and survival during development and in different cancers^20^. Since we found that SPRY2 regulates MET levels, we examined whether silencing SPRY2 or MET leads to altered cell death and proliferation in RMS. Evaluation of Cyclin D1 levels in MET depleted RD or SJRH30 cells (Supplementary Figure 2a, b and e, f) indicate that cell proliferation is not significantly impaired, suggesting that additional pathways regulate proliferation in RMS. However, Cyclin D1 levels were reduced significantly in SPRY2 depleted RD but not SJRH30 cells (Supplementary Figure 2a, b and e, f), indicating that SPRY2 may modulate pathways controlling cell proliferation in ERMS, which has higher SPRY2 levels, differently than in ARMS. Cleaved Caspase-3 measuring cell death showed a slight, statistically insignificant increase in RD cells and a significant decrease in SJRH30 cells upon SPRY2 depletion (Supplementary Figure 2c, d and g, h). Cell death was significantly increased upon MET depletion in RD but was unaffected in SJRH30 cells (Supplementary Figure 2c, d and g, h). Overall, MET or SPRY2 depletion affected cell death and proliferation differently in ERMS and ARMS, highlighting the intrinsic differences between the RMS sub-types.

### SPRY2 and MET are required to increase migratory and clonogenic potential and inhibit differentiation in RMS

**Fig. 4:**
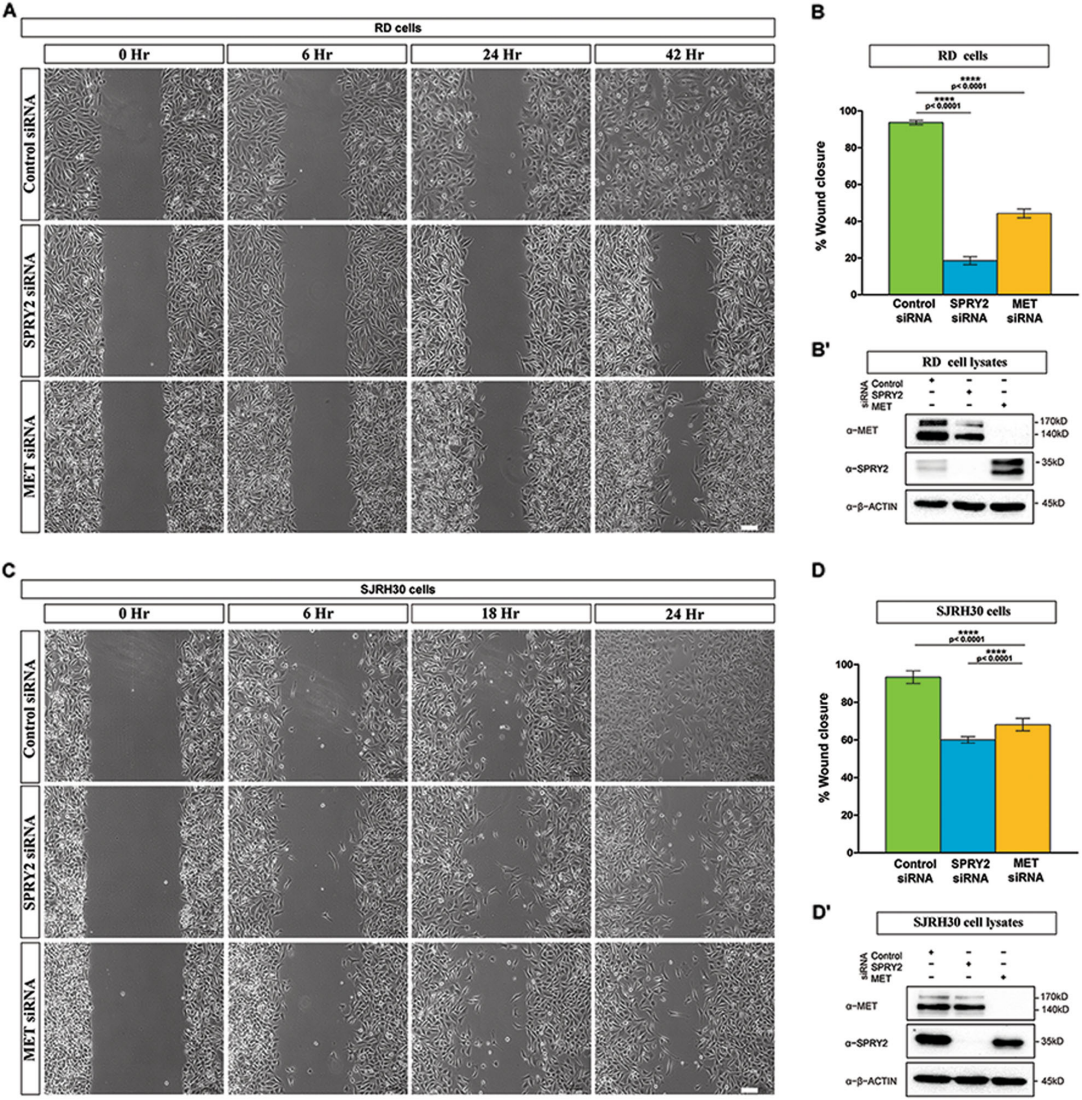
Knockdown of SPRY2 significantly reduces RMS cell migratory potential recapitulating the effect of MET downregulation. Representative bright field micrographs of wound closure assays on RD (**a**) and SJRH30 (**c**) RMS cells imaged at specific time points after ~96-h of transfection with MET or SPRY2 siRNAs, compared to those transfected with control siRNA. Scale bar is 100 µm. Bar graphs summarizing percent wound closure in the monolayers of RD (**b**) and SJRH30 (**d**) cells indicates that wound closure is significantly decreased in MET and SPRY2 siRNA transfected RMS cells when compared to control siRNA transfected cells. The graphical data represent the mean ± SEM of a minimum of 5 independent experiments. Representative western blots using cell lysates prepared at the end of the scratch assay show that MET (upper panels) and SPRY2 (middle panels) knockdown is maintained in RD (**b**′) and SJRH30 (**d**′) cells until after the completion of wound healing assay

**Fig. 5:**
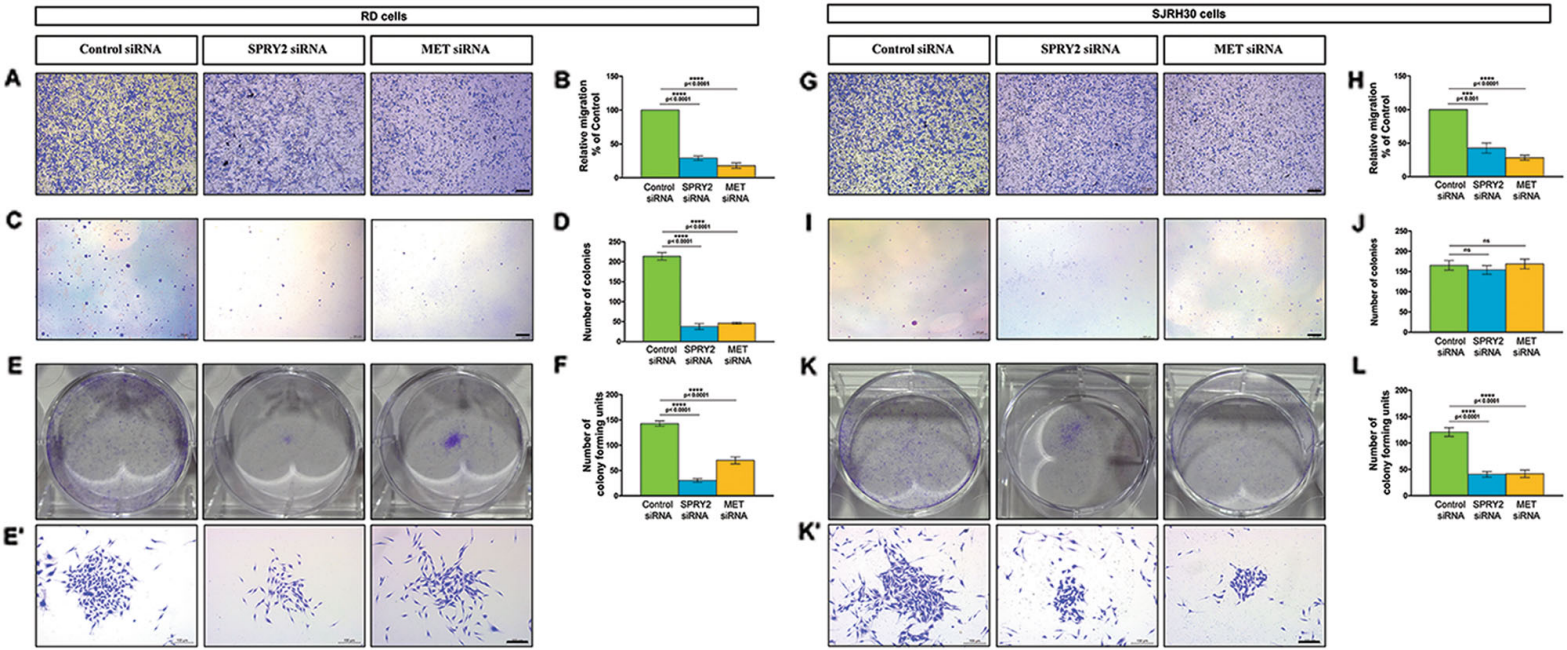
Met and SPRY2 are required for increased metastatic potential and clonogenic capacity in RMS cells. Representative images of MET, SPRY2 and control siRNA treated RD (**a**) and SJRH30 (**g**) cells allowed to migrate for 24-h in transwell migration assays are shown. Migration calculated as percentage relative to control siRNA transfected RD (**b**) and SJRH30 (**h**) cells was significantly reduced in MET or SPRY2 silenced cells. To assess the effect of MET or SPRY2 silencing on anchorage-independent colony formation, soft agar assay was performed with RD (**c**) and SJRH30 (**i**) cells PsiRT, culturing the cells for about 2-weeks. The number of colonies formed by MET or SPRY2 silenced RD cells was significantly reduced (**d**) but was not affected in the case of SJRH30 cells (**j**). Anchorage-dependent clonogenic assay was performed by culturing MET, SPRY2 or control siRNA transfected RMS cells for 8 days and staining the colonies with crystal violet (**e**,** k**). Representative images of wells and single colonies of MET, SPRY2 or control transfected RD (**e**,** e**′) and SJRH30 (**k**,** k**′) cells are shown. Colony formation was inhibited significantly in MET or SPRY2 silenced RD (**f**) and SJRH30 (**l**) cells compared to control siRNA transfected cells. The graphical data represent the mean ± SEM of a minimum of three independent experiments. Scale bar in panels **a**,** g**, **e**′ and **k**′ is 100 µm, and is 800 µm in (**c**) and (**i**)

Since SPRY2 interacts with and stabilizes MET, we studied the effect of SPRY2 or MET silencing on RMS cell migration, clonogenic potential and differentiation. We assessed the migratory potential of SPRY2 or MET depleted RD and SJRH30 cells by wound healing assay^41^. As compared to control (Fig. 4a, top panel), MET (Fig. 4a, bottom panel) or SPRY2 (Fig. 4a, middle panel) depleted RD cells showed significantly reduced percentage of wound closure (Fig. 4b) over the assay duration. Similarly, cell motility was significantly compromised in SPRY2 or MET depleted SJRH30 cells (Fig. 4d). Thus, MET (Fig. 4a, c, bottom panels) or SPRY2 (Fig. 4a, c, middle panels) silenced RD and SJRH30 cells failed to close the wound 42 and 24-h respectively after scratching the monolayer (Fig. 4a, c, top panels). End point immunoblotting validated that SPRY2 and MET remained silenced during the assay (Fig. 4b′, d′). Hence, depleting SPRY2 impaired motility in ERMS and ARMS cells (Fig. 4), mimicking the effect of MET knockdown. We validated these findings using a transwell migration assay where the percentage migration in SPRY2 or MET depleted RD (Fig. 5a, b) and SJRH30 (Fig. 5g, h) cells was significantly impaired.

Next, we assessed the effect of silencing SPRY2 or MET on the clonogenic potential of RMS cells by colony forming assays. Silencing SPRY2 or MET significantly inhibited attachment-independent clonal growth in RD cells (Fig. 5c, d) but not in SJRH30 cells (Fig. 5i, j). This disparity between the RMS sub-types may be due to their differential levels of MET, and inability of siRNA-mediated knockdown to maintain MET downregulation over the assay duration of ~2-weeks in ARMS cells with higher MET expression. Therefore, we examined the clonogenic potential of SPRY2 and MET silenced RMS cells employing adherence-dependent colony formation assay, where cells were cultured for a shorter, ~1-week duration. SPRY2 and MET were required for clonal growth of RMS cells, and the number and size of colonies markedly reduced in RD (Fig. 5e–e′ and f) and SJRH30 (Fig. 5k–k′ and l) cells upon SPRY2 or MET knockdown. These findings indicate that SPRY2 and MET regulate metastatic and clonogenic potential in RMS cells.

**Fig. 6:**
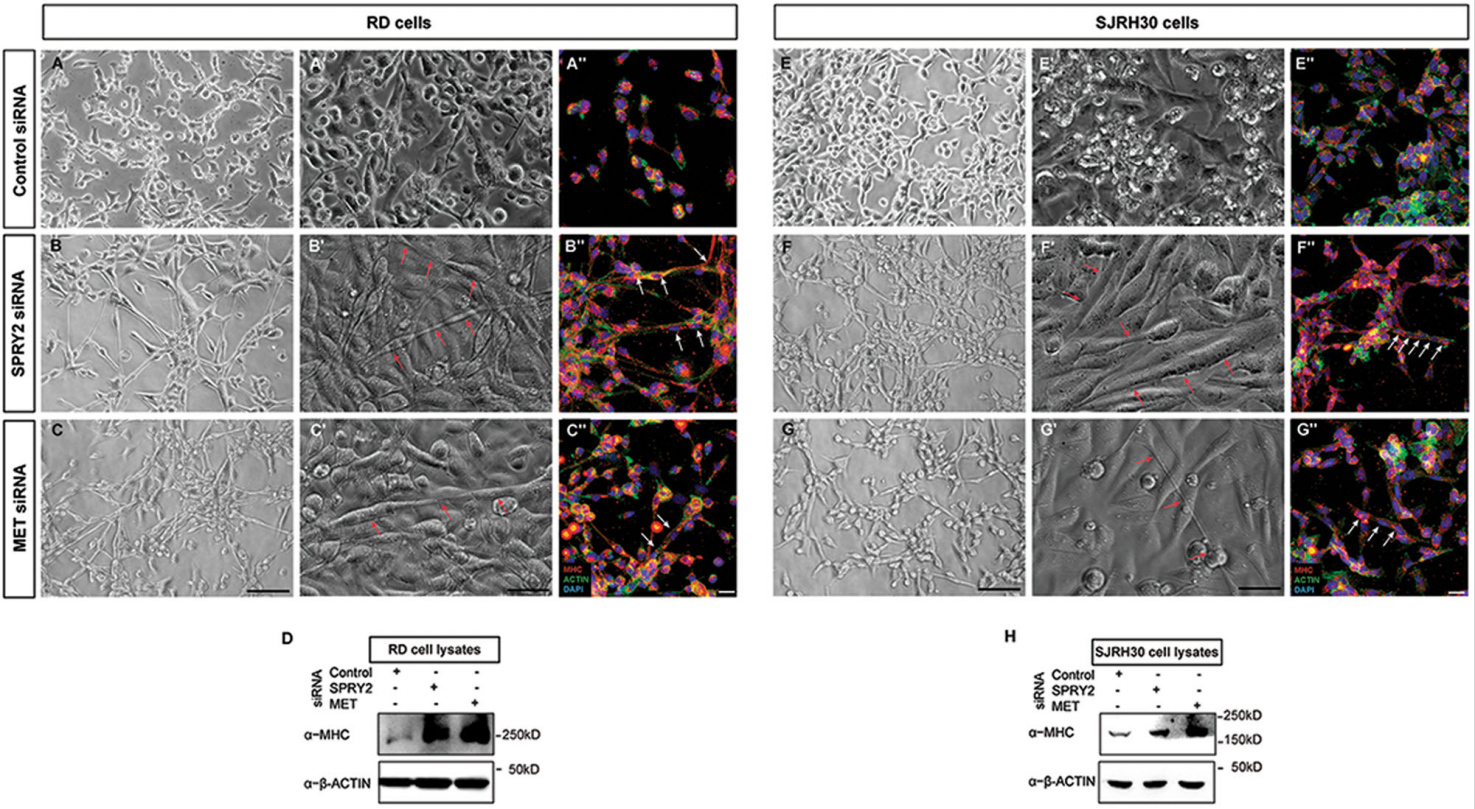
Knocking down SPRY2 or MET induces RMS cell differentiation. Representative bright field photomicrographs showing elongated, multinucleate myofiber like structures, marked with red arrows, in RD (**b**,** b**′,** c**, and **c**′) and SJRH30 (**f**,**f**′,**g** and **g**′) cells transfected with SPRY2 and MET siRNAs, compared to control cells (**a**,** a**′,** e**, and **e**′). The cells were imaged at 144-h PsiRT. This was further validated by immunofluorescence for myosin heavy chain (MHC) on RMS cells transfected with MET (**c**″,** g**″), SPRY2 (**b**″, **f**″), or control (**a**″, **e**″) siRNAs. Myofiber like structures with nuclei arranged linearly (marked with white arrows) were induced by downregulation of SPRY2 or MET in RD (**b**″,**c**″) and SJRH30 (**f**″,** g**″) cells, where MHC is labeled in red, F-actin marking the actin cytoskeleton in green and nuclei stained with DAPI in blue. These fiber like structures were absent in control siRNA transfected RD (**a**″) and SJRH30 (**e**″) cells. Scale bar in panels (**c**, **g**) is 100 µm, (**c**′,**g**′) is 50 µm and (**c**″,** g**″) is 25 µm. Immunoblots of MET or SPRY2 siRNA transfected RD (**d**) and SJRH30 (**h**) cell lysates, prepared at 144-h post transfection, show increased MHC expression as compared to control siRNA transfected cells. This substantiates the phenotypic differentiation observed in RD and SJRH30 cells seen by bright field and immunofluorescent imaging

Constitutive activation of MET inhibits differentiation while shRNA-mediated downregulation of MET induces differentiation in RMS^42^. Therefore, we sought to determine whether depleting SPRY2, which reduces MET levels, could induce RMS cell differentiation. Consistent with earlier reports, MET knockdown induced RD (Fig. 6c–c′) and SJRH30 (Fig. 6g-g′) cells to change morphology, becoming elongated, multi-nucleate, and compared to controls myofiber-like (Fig. 6a–a′ and e–e′). Remarkably, SPRY2 depleted RD (Fig. 6b-b′) and SJRH30 (Fig. 6f–f′) cells formed similar differentiated structures (red arrows) by 144-h PsiRT. Thus, SPRY2 or MET depletion in RMS cells overcomes the differentiation-block, forming myofiber-like structures that are multi-nucleate, with linearly arranged nuclei characteristic of myofibers^43^, and positive for MHC-EMBRYONIC, a myogenic differentiation marker (Fig. 6b″, c″, f″, g″). MHC-EMBRYONIC expression was observed in control RD (Fig. 6a″) and SJRH30 (Fig. 6e″) cells as reported previously^11^. However, compared to control cells, MHC-EMBRYONIC was expressed at relatively higher levels in MET or SPRY2 depleted RMS cells 144-h PsiRT (Fig. 6d, h). Taken together, this indicates that silencing SPRY2, similar to MET downregulation, promotes differentiation in RMS cells.

### SPRY2 and MET promote ERK/MAPK signaling in RMS

MAPK signaling regulates cell migration, motility and differentiation^44–50^. Since MET is known to regulate both ERK/MAPK and p38/MAPK, we hypothesized that reduced migration and induction of differentiation upon MET or SPRY2 depletion in RMS could be mediated by these pathways^20^. Therefore, we assayed the activation of these two MAPK signaling branches in RMS, 144-h PsiRT, by western blotting.

**Fig. 7:**
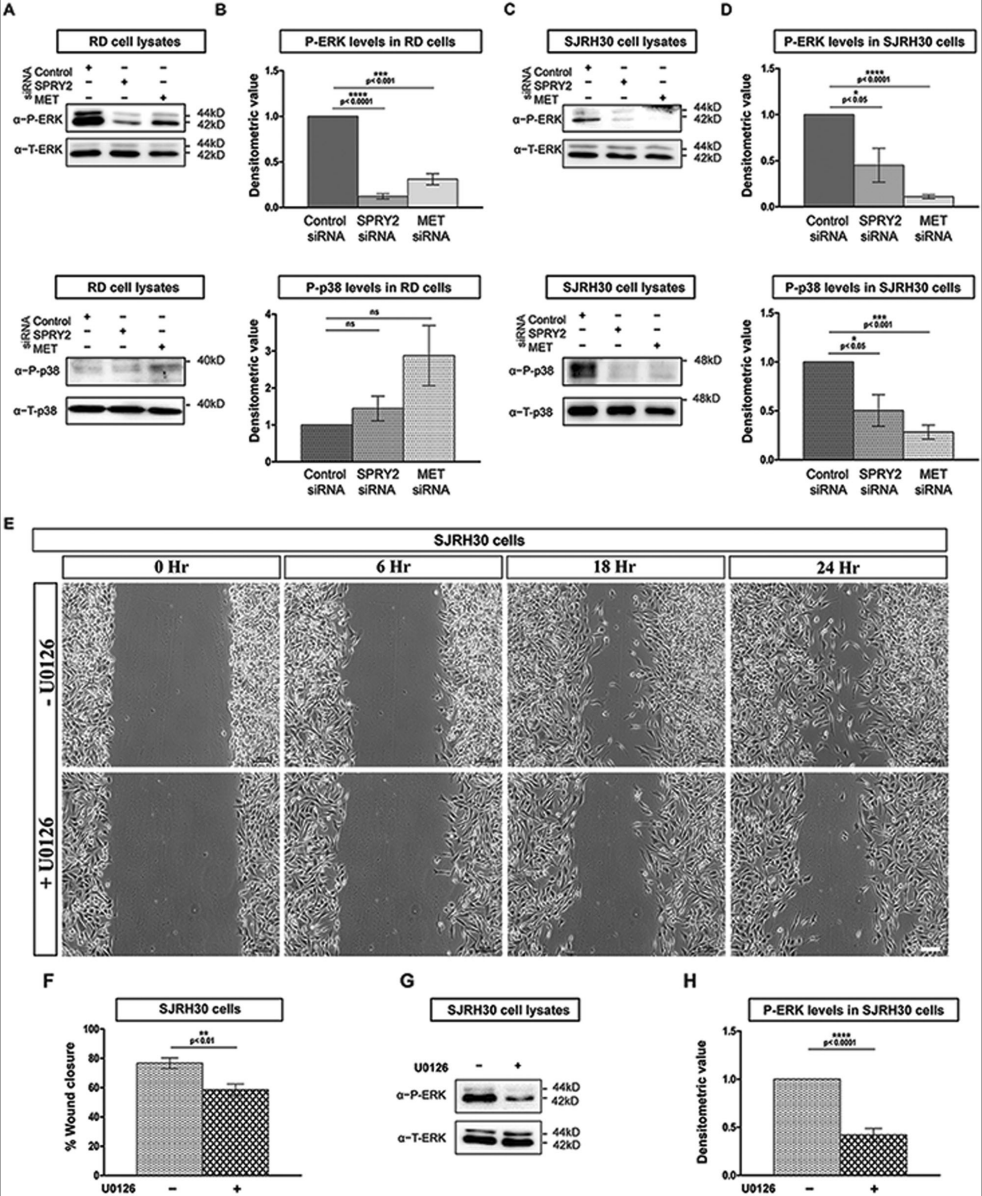
MET or SPRY2 knockdown causes similar alterations in MAP Kinase pathway activity in RMS cells. RMS cells were cultured for 144-h post transfection with MET, SPRY2, or control siRNA and lysates were separated by SDS-PAGE and analyzed by western blotting for MAP kinase pathway activation. Representative blots for levels of phosphorylated and total ERK1/2 (upper panels), as well as phosphorylated and total p38 (lower panels) detected using specific antibodies in RD (**a**) and SJRH30 (**c**) cells are shown. The ratios of pERK1/2 to T-ERK1/2 and p38 to total p38 were measured by densitometry (**b**,** d**). ERK activation is significantly reduced in both MET and SPRY2 silenced RD (**b**, upper panel) and SJRH30 (**d**, upper panel) cells when compared to control cells. However, phosphorylation of p38 is unchanged in RD cells (**b**, lower panel) but significantly reduced in SJRH30 cells (**d**, lower panel) between MET/SPRY2 and control siRNA transfections. Representative bright field micrographs of wound closure assays using SJRH30 cells, imaged at specific time points after incubation with (+) or without (–) the MEK/ERK inhibitor U0126 (**e**). Bar graphs show that wound closure in the monolayers of SJRH30 is significantly decreased in U0126 treated cells compared to untreated cells (**f**). Representative western blots for p-ERK1/2 and total-ERK1/2 using cell lysates prepared at the end of the wound closure are shown (**g**). Densitometry shows that p-ERK1/2 levels are significantly lower in U0126 treated SJRH30 cells when compared to untreated cells at the end of scratch assay (**h**). The graphical data are presented as mean ± SEM of a minimum of three independent knockdown experiments. Scale bar in **e** is 100 µm

We found that ERK/MAPK activation was significantly diminished in SPRY2 or MET depleted RD (Fig. 7a, b, upper panels) and SJRH30 (Fig. 7c, d, upper panels) cells. Interestingly, the inhibition of ERK/MAPK activation in RMS cells correlated with induction of differentiation observed at 144-h PsiRT. No significant difference in p38/MAPK phosphorylation in MET or SPRY2 silenced RD cells was observed (Fig. 7a, b, lower panels). Thus, in RD cells, weakening of ERK/MAPK activation downstream of MET is sufficient to promote differentiation. In contrast, silencing MET or SPRY2 in SJRH30 cells significantly decreased p38/MAPK activation (Fig. 7c, d, lower panels). These findings indicate that although the phosphorylation status of p38/MAPK in ERMS and ARMS are different, highlighting the RMS sub-type specific requirement of p38 activity, the effect of MET and SPRY2 depletion is identical. This is similar to the requirement of p38 activation during early stages and its inhibition at late stages of myogenesis reported previously^47,49^.

To test whether reduced MAPK signaling resulting from silencing SPRY2 or MET is responsible for their impaired migratory potential, we assessed SJRH30 cell migration upon treatment with the MEK/ERK inhibitor U0126. Inhibition of MAPK signaling significantly impaired cell motility (Fig. 7e, f) and end point phosphorylated ERK levels showed that inhibition was efficiently maintained (Fig. 7g, h). Overall, SPRY2 engages with the MET receptor and stabilizes it, functioning to maintain downstream ERK signaling to sustain RMS migratory potential and prevent differentiation in ERMS and ARMS.

## Discussion

MET is a RTK known to be dysregulated in RMS, demonstrated to be a promising therapeutic candidate^18,19,27,51^. However, the reasons underlying mis-regulation of MET in RMS are incompletely understood. Using embryonal and alveolar RMS cell lines, we show for the first time that SPRY2 interacts with and stabilizes MET to sustain downstream signals that maintain the migratory and clonogenic potential and block differentiation in RMS.

We found that MET transcript and protein levels varied across RMS cell lines but were higher in ARMS compared to ERMS cells, which possibly correlates with its heightened aggressiveness and metastatic potential^19,26,38,42^. Although HGF-MET signaling induced SPRY2 expression in leiomyosarcoma^35^, we found that SPRY2 levels were higher in ERMS cells that have low MET expression, compared to ARMS cells, suggesting additional mechanisms regulate SPRY2.

SPRY2 modulates MET signaling in cancer types such as leiomyosarcomas, hepatocarcinomas, and colonic adenocarcinomas^34–36^. MET is overexpressed in non-small cell lung carcinoma (NSCLC), where miR-27a binds and downregulates MET and SPRY2 transcripts^52^. Intriguingly, downregulation of MET protein required concomitant overexpression of miR-27a and silencing of SPRY2, indicating that while miR-27a controls MET transcript levels, SPRY2 is critical in stabilizing MET protein in NSCLC^52^. SPRY2 is known to positively regulate MET levels in colon carcinoma, although it is unclear whether SPRY2 induces MET transcriptional activation or reduces MET degradation^36^. Thus, SPRY2 functions to maintain MET levels in multiple tumor types, although the precise mechanism is unclear. We observed that MET protein was significantly downregulated in SPRY2 depleted RMS cells, without altering MET transcript levels. This suggests that at least in RMS, SPRY2 does not regulate MET expression transcriptionally, but presence of SPRY2 inhibits MET protein degradation resulting in its stabilization. SPRY2 knockdown resulted in MET degradation preferentially via the proteasomal pathway in ERMS and lysosome-mediated pathway in ARMS, indicating that multiple degradatory mechanisms regulate MET, as reported previously^39^. Since we found that MET and SPRY2 interact with each other, it is likely that this interaction results in MET stabilization. Interestingly, this interaction also stabilizes SPRY2, since MET depletion led to a reduction in SPRY2 protein levels without affecting transcript levels in ARMS. One possibility is that the MET-SPRY2 interaction renders motifs on both proteins that target them for degradation, such as ubiquitination, inaccessible.

Although there are differences between embryonal and alveolar RMS, both tumor sub-types share similar features such as tumor cells exhibiting muscle cell characteristics and inhibition of differentiation^9–12^. Previous studies indicate that inhibition of MET reduces the metastatic potential and overrides the differentiation block in RMS cells^26,27^. Our finding that SPRY2 stabilizes MET, and depletion of SPRY2 leads to MET downregulation indicates that loss of SPRY2 should lead to decreased metastatic potential and permit differentiation of RMS cells. We found this to be the case where SPRY2 depletion led to decreased migration, and induction of differentiation. Also, SPRY2 and MET conferred clonogenic potential on RMS cells and silencing either significantly reduced colony forming ability of these cells. Whether SPRY2 plays MET independent roles in regulating RMS migration, differentiation or clonogenic potential is unknown, and could be investigated by overexpressing SPRY2 in MET depleted RMS cells.

We did not observe any effect of MET silencing on cellular proliferation in RMS cells. A previous study reported that RMS proliferation rate decreased upon downregulation of MET^27^. We observed a significant increase in cell death in ERMS cells upon MET knockdown, but not in ARMS, compared to a study which reported increased cell death in both ERMS and ARMS^27^. This could be due to differences in experimental set up, where we analyzed cells six days PsiRT, whereas the previous study used a doxycycline-inducible conditional lentiviral shRNA construct targeting MET over five days to study proliferation and cell death^27^. We found that downregulation of SPRY2 caused significantly reduced proliferation in ERMS and significantly decreased cell death in ARMS, indicating that SPRY2 might have MET independent functions in RMS. The varying trends of cell death and proliferation seen in MET or SPRY2 silenced RD and SJRH30 cells in this study reflect the intrinsic molecular differences between the two RMS sub-types.

Two pathways reported to function downstream of MET are the ERK (p44/42)/MAPK and p38/MAPK pathways^20^. While the ERK (p44/42)/MAPK pathway regulates cell migration downstream of MET receptor, both MAPK pathways are essential for myogenic differentiation^44–49^. Inhibition of ERK/MAPK signal is required for the myogenic program to proceed normally, whereas p38 activation is required at early stages but needs to be suppressed at later stages for myogenic differentiation^46,47,49^. Our results suggest that ERK/MAPK pathway is the direct target of MET and SPRY2 in RMS, and the decreased metastatic potential and induction of differentiation observed upon MET or SPRY2 depletion is mediated by this pathway. Interestingly, we also observed a significant decrease in p38/MAPK activation in ARMS but not ERMS cells upon MET or SPRY2 depletion, which might be because ARMS tumor cells resemble the developing fetal muscles compared to ERMS which exhibit characteristics of embryonic muscles^11^. At later stages of myogenesis, p38/MAPK is suppressed, which may not be taking place in ARMS cells, contributing to the lack of differentiation^49^. ERMS cells migrated at a slower rate (42-h) compared to ARMS cells (24-h) in wound-healing assays, agreeing with reports that ARMS is relatively more metastatic and aggressive compared to ERMS^26,38^.

**Fig. 8:**
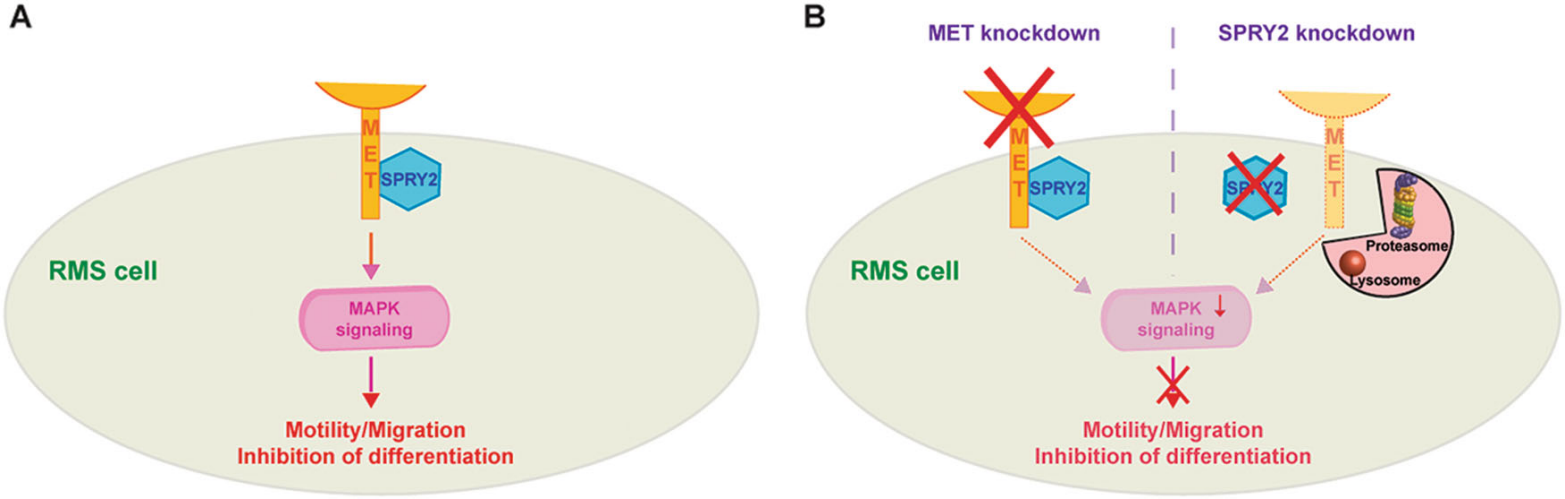
SPRY2 is required to stabilize and regulate signaling downstream of the MET receptor. MAPK signaling transduced by aberrant MET activation and sustained by SPRY2 mediated stabilization of the MET receptor inhibits myogenic differentiation and confers metastatic characteristics on RMS cells (**a**). Silencing MET induces differentiation and reduces the metastatic potential of RMS cells by dampening the MAPK signaling (**b**). Silencing SPRY2 decreases MET stability and results in reduced MAPK signaling, thereby recapitulating the effects of MET silencing in RMS cells (**b**). Proteasomal degradation is the preferred pathway of MET degradation in ERMS (RD) cells while lysosome mediated degradation is the preferred pathway of MET degradation in ARMS (SJRH30) cells. MET degradation by these pathways is prevented when SPRY2 is bound to MET

We find that in addition to MET, SPRY2, a bimodal regulator of RTK signaling also exhibits elevated levels of expression in RMS. However, SPRY2 has never been shown to directly interact with the RTK itself to regulate it or downstream signaling. Here, we show that SPRY2 colocalizes and interacts with MET, stabilizing and preventing MET degradation, which leads to elevated ERK/MAPK signaling (Fig. 8). All of this facilitates the maintenance of increased migratory potential and inhibition of differentiation, characteristic of RMS (Fig. 8). The identification of this unique molecular interaction between SPRY2 and MET that functions to regulate the oncogenic properties of RMS raises the possibility of leveraging it for future therapeutic interventions in RMS and other tumor types where MET is aberrantly activated.

## Materials and Methods

### Cell culture

Human RMS cells of embryonal (RD; Cat# CCL-136) and alveolar (SJRH30; Cat# CRL-2061) sub-type were purchased from American Type Cell Culture (ATCC) (Manassas, VA, USA). ARMS cell lines - RH4, RH28 and RH41 were kindly provided by Dr. Peter J. Houghton (Nationwide Children’s Hospital, Columbus, Ohio, USA). These cell lines were grown and maintained as recommended, in Dulbecco Modified Eagle Medium (DMEM) (Gibco, Thermo Fisher Scientific, Waltham, MA, USA; Cat# 11995065) supplemented with 10% (v/v) fetal bovine serum (Sigma, St. Louis, Missouri, USA; Cat# F2442) and 1% penicillin-streptomycin (i.e., 100 U/ml, Gibco; Cat# 15140122) at 37 °C, 5% CO_2_ and 95% humidity.

For expression analyses at transcript and protein levels, ~60,000 and 3,00,000 cells were plated in each well of 24-well (Nunc, Roskilde, Denmark; Cat# 142485) and 6-well plates (Corning, New York, USA; Cat# 3506), respectively and allowed to grow to ~80% confluence. Cells from 4 wells of a 24-well plate and one 6-well plate were harvested per replicate, to isolate RNA and prepare protein lysates, respectively. These experiments were carried out in triplicates. For knockdown experiments, reverse transfection was performed in RD and SJRH30 cells. Briefly, ~60,000 cells were seeded in each well of a 24-well plate that was pre-layered with transfection mix comprising 100 µl Opti-MEM (Gibco; Cat# 31985070), 10 nM of MET or SPRY2 or control siRNAS (Silencer Select siRNAs, Ambion, Thermo Fisher Scientific; Cat# s8702, s20028 and 4390847, respectively) and 2 µl Lipofectamine RNAiMAX (Invitrogen, Thermo Fisher Scientific; Cat# 13778150). At 48 and 144-h post siRNA transfection (PsiRT), 4 wells of a 24-well plate and one 24-well plate were harvested per replicate to isolate RNA and prepare protein extracts, respectively. ON-TARGETplus SMARTpool siRNAs targeting human MET (10 nM) and SPRY2 (50 nM) (Dharmacon, Cambridge, UK; Cat# L-003156-00-0005 and L-005206-00-0005, respectively) were used to validate the knockdown observed using the Silencer Select siRNAs (Ambion). These experiments were performed a minimum of three times. All experiments requiring knockdown were subsequently performed using Ambion Silencer Select siRNAs. Where specified, RMS cells were incubated in the presence of lysosomal inhibitor Bafilomycin A1 (100 nM for 4-h, Sigma; Cat# B1793), proteasomal inhibitor MG132 (25 µM for 6-h; Cayman Chemical Company, Michigan, Ann Arbor, USA; Cat#1001268), or vehicle control, 96-h PsiRT. For immunofluorescence studies, RMS cells were seeded onto gelatin-coated coverslips (Neuvitro, Vancouver, WA, USA; Cat# GG-12) in 24-well plates (Nunc; Cat# 142485). Approximately 30,000 cells/well were plated for immunofluorescence based expression and colocalization experiments, whereas ~50,000 cells/well were reverse transfected and seeded, as described above, for analysis of knockdown induced differentiation of RMS cells.

### RNA isolation, cDNA synthesis, and qPCR

RNA was isolated from RMS cells harvested as described in the cell culture section above, using RNeasy mini kit (Qiagen, Hilden, Germany; Cat# 74106). cDNA was synthesized from 1 µg of RNA using SuperScript III reverse transcriptase (Invitrogen, Cat# 18080-044) and oligo (dT) (Invitrogen, Cat# 58862). *MET* and *SPRY2* transcripts were quantified by quantitative real time polymerase chain reaction (qPCR) using SYBR Green with ROX as internal reference (Applied Biosystems, Thermo Fisher Scientific; Cat# 4367659) using the ABI 7500 Fast Real Time PCR system (Applied Biosystems). The primers used for gene expression profiling in different RMS cell lines are listed in Supplementary Table 1. *GAPDH* and *HPRT* were used as endogenous controls or reference genes for normalizing expression of target genes (that is *MET* and *SPRY2*)^53,54^. RNA from mesenchymal stem cells, closest appropriate control for RMS cells that are thought to originate from cells of the mesenchymal origin^14^, was used as a control or calibrator. All the qPCR assays were performed in a final reaction volume of 20 μl using 100 nM forward and reverse primers of the respective genes, as recommended by ABI. Each reaction was performed in triplicates. The expression of target genes in the siRNA transfected RMS cells was normalized to the levels in control siRNA transfected cells^55^. Three replicate cDNA samples were used for quantifying endogenous expression of target genes in different cell lines. Three matched sets of *MET* or *SPRY2* and control siRNA transfected samples were used as biological replicates for performing expression analysis and the error bar represents±standard error of the mean (SEM).

### Co-immunoprecipitation

RMS cells were lysed in ice-cold lysis buffer (200 mM Tris, pH7.4; 120 mM NaCl; 0.2% sodium deoxycholate; 0.1% SDS; 1 mM EGTA, 1% Triton-X 100) supplemented with 1× protease inhibitor (Sigma; Cat# P8340). Debris was removed by centrifugation at 6000 rpm for 5 min at 4 °C and the lysates obtained were quantified using BCA Protein Assay kit (Pierce, Thermo Fisher Scientific; Cat# 23225) as per manufacturer’s protocol. Approximately 500 µg protein was incubated with Protein A Sepharose 4 Fast flow beads (GE Healthcare Life Sciences, Chicago, IL, USA; Cat# 17-5280-01) for 1-h at 4 °C on a tube mixer, followed by centrifugation to remove the beads and the proteins that were bound non-specifically. Subsequently, the pre-cleared lysates were used to immunoprecipitate MET-conjugated proteins by incubation with MET antibody overnight at 4 °C on a tube mixer. IgG raised in rabbit was used as a control antibody. The antibody-bound proteins were then incubated with Protein A Sepharose 4 Fast flow beads for 6-h at 4 °C on a tube mixer to capture the immunocomplexes. Immunoprecipitates were washed thrice using ice-cold lysis buffer and eluted by boiling in 2× Laemmli sample buffer at 95 °C for 3 min. The immunoprecipitates were subjected to western analysis as described below. Antibodies and the concentrations used for immunoprecipitation are listed in Supplementary Table 2.

### Immunoblotting

Protein lysates were prepared from RMS cells that were plated, cultured, and harvested at required time points as explained above in the cell culture section. Briefly, the cells were harvested by aspirating the medium from the wells, trypsinizing and centrifuging at 6000 rpm/4 °C/6 min. Subsequently, cells were washed with ice-cold 1 × DPBS (Gibco; Cat# 14190-144) twice, followed by centrifugation. Cells were lysed in ice-cold radioimmunoprecipitation assay (RIPA) buffer (Sigma; Cat# R0278-50) containing protease inhibitor (Sigma; Cat# P8340) and phosphatase inhibitor (Cell Signaling Technology, Danvers, MA, USA; Cat# 58705), each at 1:100 dilution. The protein samples were quantified using BCA Protein Assay kit (Thermo Fisher Scientific; Cat# 23225) as per manufacturer’s protocol. Equal concentrations of protein extracts were separated by 10% or 12% SDS-PAGE, depending on the molecular weight of the protein to be detected, followed by transfer to PVDF membrane (Millipore; Billerica, MA, USA; Cat# iPVH00010) at 4 °C overnight. Subsequently, the membranes were blocked in 5% skimmed milk for 5-h, incubated overnight in primary antibody at 4 °C, and 2-h in appropriate HRP-conjugated secondary antibody at room temperature. Signals were detected using the Luminata Forte HRP substrate (Millipore; Cat# WBLUF0100). Blots were imaged using ImageQuant LAS 4000 and densitometric analyses were performed using and ImageQuant TL v7.0 software (GE Healthcare Life Sciences). For densitometric analyses, reference genes used as loading controls for normalization were β-Actin, GAPDH, or β-Tubulin. Data from a minimum of three independent replicates were plotted as an average, with the error bars representing ± SEM. Antibodies and the concentrations used for western blotting are listed in Supplementary Table 2.

### Wound closure and transwell migration assays

RMS cells were seeded in 6-well plates and reverse transfected with MET, SPRY2, or control siRNAs respectively, as described above in the cell culture section. A seeding cell density of ~2.5 × 10^5^ cells/well was used so that the cells attain about 90% confluence by around 96-h post siRNA transfection. Subsequently, the media containing the transfection mix was aspirated out of each well, preserved in labeled tubes and the cell monolayers were scratched carefully with a sterile 200 μl pipette tip. The wells were washed gently with 1×DPBS (Gibco; Cat# 14190-144) thrice to get rid of the scraped floating cells and the preserved transfection mix was added back to the respective wells. The scratch was imaged immediately for the zero-time point (0) using an inverted microscope (Nikon Eclipse TS100, Minato, Tokyo, Japan). Subsequently, the cells were incubated at 37 °C, 5% CO_2_ and 95% humidity, and the scratch monitored and documented over time at the same positions. A mark was made on the plastic plate as a reference point to ensure that the same area was imaged every time over the time course of the experiment^41^. Imaging was performed until the time point (t) when the wound closed in the control siRNA transfected RMS cells. The images were used to measure the scratched area at zero-time point (A_0_) and at the time point of closure (A_t_), using a macro (freely available at http://rsb.info.nih.gov/ij/) written at Montpellier RIO Imaging^56^ for ImageJ software^57,58^. Percentage wound closure was calculated using the formula ((A_0_ - A_t_)/A_0_)×100. Wound closure assays were also performed on SJRH30 cells in the presence and absence of MEK/ERK inhibitor U0126 (Cell Signaling Technology; Cat# 9903). The wound healing assays were performed independently a minimum of five times for each transfection.

Transwell migration assay was performed to evaluate the effect of MET and SPRY2 knock-down on metastatic potential of RMS cells. Briefly, 96-h post transfection with MET, SPRY2 or control siRNA, RMS cells were trypsinized and re-suspended in serum free medium. Approximately 80,000 RD and 60,000 SJRH30 cells were seeded in transwell inserts (8 µm pore size, Corning; Cat#3464), and placed into 24-well cell culture plates containing DMEM with 10% FBS. After incubation at 37 °C for 24-h, non-migrating cells on the upper surface of the membrane were removed with cotton swabs and migrated cells at the base of the inserts were fixed in 4% PFA (~5 min), permeabilized with methanol (20 min) and stained with 2% crystal violet dye (15 min). Cells were counted manually and imaged using a stereomicroscope (Leica S8 APO, Houston, Texas, USA). Percentage cell migration was calculated relative to control siRNA transfected RMS cells. The transwell migration assays were performed independently a minimum of three times for each transfection. Graphical data for the wound closure and transwell migration assays are represented as mean ± SEM.

### Clonogenic assays

Anchorage-independent colony forming ability of MET or SPRY2 silenced RMS cells was examined by soft agar assay. A base layer was prepared by pipetting 2 ml 0.7% agar (BD Bacto agar, Fisher Scientific; Cat# 214010) in 10%FBS/1%Pen-Strep/DMEM, in 6-well plates. This base layer was overlaid with 0.35% agar with 6000 siRNA transfected RMS cells (96-h PsiRT) suspended in it. The cells were then cultured for about 2-weeks. 1 ml culture medium was supplemented to each well every 5 days to prevent desiccation of agar. Colonies were fixed and stained using 0.05% crystal violet in neutral buffered formalin for 1-h. Colonies were counted manually and imaged using a stereomicroscope (Leica S8 APO).

For anchorage-dependent colony formation assay MET, SPRY2, or control siRNA transfected RMS cells were plated 96-h PsiRT in 6 well plates at 3000 cells/well and cultured for 8 days. Colonies were washed twice with 1×PBS, fixed in ice cold methanol (20 min) and stained with 2% crystal violet (~30 min). The wells were imaged using a digital camera and representative individual colonies form different transfections were imaged using a stereomicroscope (Leica S8 APO). Colony forming units or colonies comprising >30 cells were counted manually under the stereomicroscope.

Both the clonogenic assays were performed independently a minimum of three times for each transfection and graphical data is represented as mean ± SEM.

### Immunofluorescence and microscopy

RMS cells for immunofluorescence were grown as detailed in the cell culture section above. The cells were fixed in 4% paraformaldehyde (PFA) for 20 min and washed with PBS following which they were blocked with 5% goat serum (BioAbChem, Ladson, SC, USA; Cat# 72-0480) in PBS containing 0.1% Triton-X-100 for 1-h at room temperature (RT). The cells were incubated overnight at 4 °C with appropriate concentrations of respective primary antibodies listed in Supplementary Table 2, washed thrice with PBS, incubated with the appropriate secondary antibody for 2-h at RT, and washed with PBS. In experiments where signal amplification was required for detection of the target protein, cells were incubated with a biotin conjugated secondary antibody for 2-h at RT and then with fluorophore conjugated to streptavidin for 1-h at RT, washed thrice with PBS, rinsed in distilled water and allowed to dry. Coverslips were mounted with DAPI Fluoromount-G (Southern Biotech, Birmingham, AL, USA; Cat# 0100-20). Images were captured using the Leica TCS SP5 or SP8 confocal microscopes using identical settings for a particular staining across treatments for all replicates.

The colocalization between SPRY2 and MET was quantified using the colocalization tool in the Leica Application Suite X (LAS X) software, with the threshold and background parameters for both channels set to 60% and 20%, respectively. Analysis was performed on 17 individual cells, in three different images, showing SPRY2-MET colocalization and the mean Pearson’s correlation coefficient was calculated from these regions of interest (ROIs)^40^.

### Statistical analyses

Experimental data was plotted in GraphPad Prism 5 (GraphPad Software Inc., CA, USA) and analyzed using the parametric, paired t-test using the SigmaPlot12.5 (Systat Software Inc., Germany). All graphical data is represented as mean ± SEM, and each experiment was performed at least in triplicates. The *p*-value is indicated on the graph along with asterisks and *p*-value ≤ 0.05 is considered significant. The level of significance is indicated as *p < 0.05; **p < 0.01; ***p < 0.001; ****p < 0.0001.

## Electronic supplementary material

Supplementary Figure 1

Supplementary Figure 2

Supplementary Table 1

Supplementary Table 2
