# Supplementary figures and images for: SPRY2 is a novel MET interactor that regulates metastatic potential and differentiation in rhabdomyosarcoma
Source: Cell Death Dis. 2018 Feb 14;9(2):237. doi: 10.1038/s41419-018-0261-2 (PMC5833614; doi:10.1038/s41419-018-0261-2)

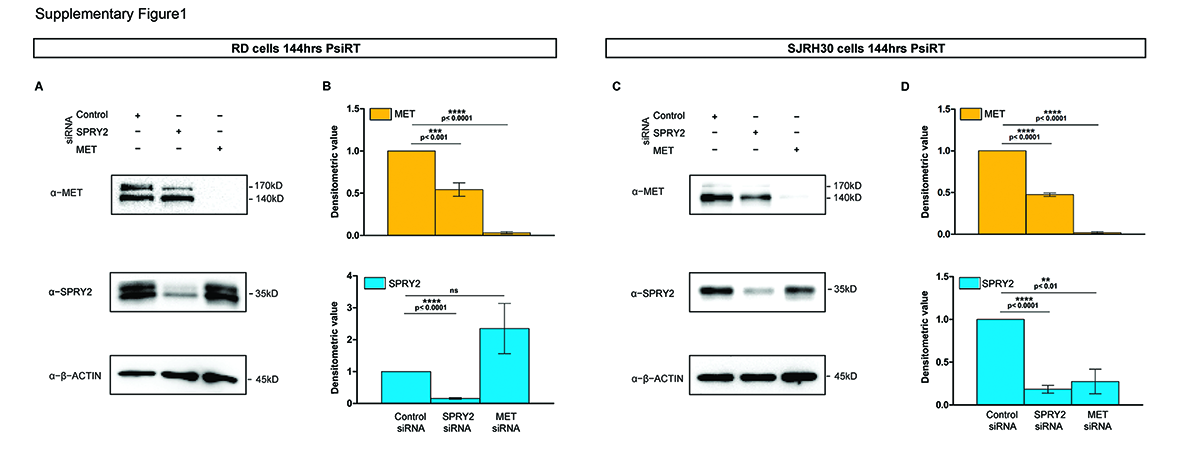

Supplement: Supplementary file 1 — Supplementary Figure 1 [file 41419_2018_261_MOESM1_ESM.tif]

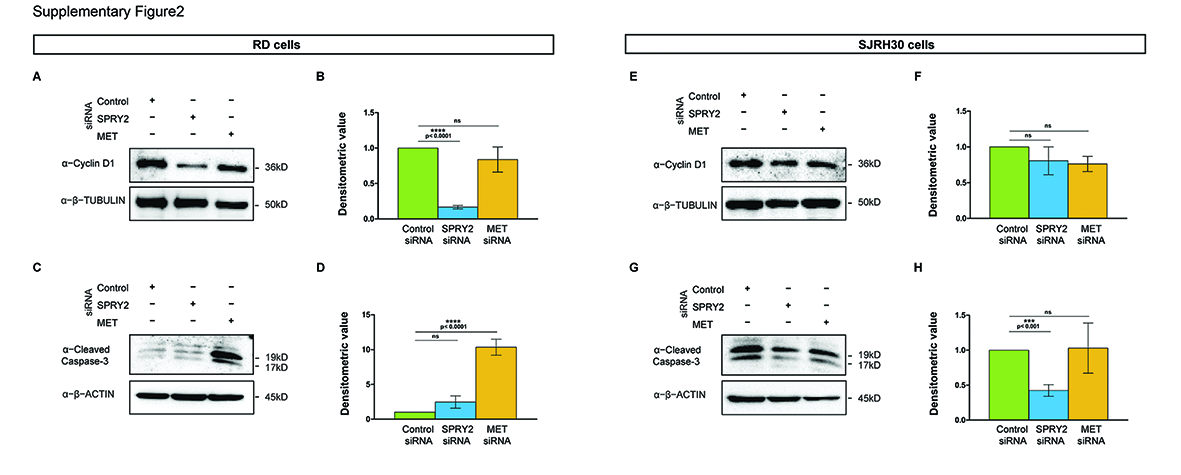

Supplement: Supplementary file 2 — Supplementary Figure 2 [file 41419_2018_261_MOESM2_ESM.tif]
