# Supplementary material for: SPRY2 is a novel MET interactor that regulates metastatic potential and differentiation in rhabdomyosarcoma
Source: Cell Death Dis. 2018 Feb 14;9(2):237. doi: 10.1038/s41419-018-0261-2 (PMC5833614; doi:10.1038/s41419-018-0261-2)
Supplement: Supplementary file 3 — Supplementary Table 1 [file 41419_2018_261_MOESM3_ESM.docx]

**Supplementary Table 1. Primers used for RT-qPCR analysis**

| **Gene** | **Direction** | **Primer sequence 5’ to 3’** | **Location** | **Product size (bp) using cDNA template** |
| --- | --- | --- | --- | --- |
| *MET* | Forward  Reverse | TGGACAATGATGGCAAGAAAAT  CTTCACTTCGCAGGCAGAT | Exon16  Exon19 | 159bp |
| *SPRY2* | Forward  Reverse | AGCAGAGGTTGGAAAGAAA  CCAGCAGGCTTAGAACACATC | Exon 1  Exon 2 | 141bp |
| *GAPDH* | Forward  Reverse | GCCACATCGCTCAGACACCAT  ACCAGGCGCCCAATACG | Exon1  Exon2 | 72bp |
| *HPRT* | Forward  Reverse | TGACACTGGCAAAACAATGCA  GGTCCTTTTCACCAGCAAGCT | Exon6  Exons6 and 7 | 94bp |
