# Supplementary material for: SPRY2 is a novel MET interactor that regulates metastatic potential and differentiation in rhabdomyosarcoma
Source: Cell Death Dis. 2018 Feb 14;9(2):237. doi: 10.1038/s41419-018-0261-2 (PMC5833614; doi:10.1038/s41419-018-0261-2)
Supplement: Supplementary file 4 — Supplementary Table 2 [file 41419_2018_261_MOESM4_ESM.docx]

**Supplementary Table 2. Antibodies used for western blots, co-immunoprecipitation and immunofluorescence**

| **Antibody** | **Type** | **Source** | **Product No.** | **Working Concentration (µg/ml)** |
| --- | --- | --- | --- | --- |
| **Primary antibodies** | | | | |
| MET | Rabbit IgG | Cell Signaling Technology | 8198 (D1C2) | 0.03 (WB); 0.17 (IF); 1.0 (Co-IP) |
| SPRY2 | Mouse IgG1κ | Sigma | WH00010253M1 (1E10) | 0.67 (WB); 2.5 (IF) |
| Phospho-p42/44 MAPK (Thr202/Tyr204) | Rabbit IgG | Cell Signaling Technology | 4370 (D13.14.4E) | 0.25 (WB) |
| p42/44 MAPK (Erk1/2) | Rabbit IgG | Cell Signaling Technology | 4695 (137F5) | 0.04 (WB) |
| Phospho-p38 MAPK (Thr180/Tyr182) | Rabbit IgG | Cell Signaling Technology | 4511 (D3F9) | 0.04 (WB) |
| p38 MAPK | Rabbit | Cell Signaling Technology | 9212 | 0.01 (WB) |
| Cyclin D1 | Rabbit IgG | Cell Signaling Technology | 2978 | 0.08 (WB) |
| Cleaved Caspase-3 | Rabbit IgG | Cell Signaling Technology | 9664 | 0.11 (WB) |
| β-Actin | Mouse IgG2b | Cell Signaling Technology | 3700 (8H10D10) | 0.24 (WB) |
| GAPDH | Rabbit IgG | Bethyl Laboratories Inc. | A300-640A | 0.2 (WB) |
| β-Tubulin | Mouse IgG2b | Sigma | T5076 (SDL.3D10) | 2.0 (WB) |
| MyHCemb | Mouse IgG1 | Developmental Studies Hybridoma Bank | F1.652 - sera | 1:2 (IF); 1:10 (WB) |
| Normal Rabbit IgG | Rabbit IgG | MERCK | 12-370 | 2.0 (Co-IP) |
| **Secondary antibodies, fluorescent coupled conjugates** | | | | |
| Cy3 conjugated Goat anti-mouse | Goat IgG | Jackson ImmunoResearch Laboratories | 115-165-146 | 7.5 (IF) |
| Cy3 conjugated Goat anti-rabbit | Goat IgG | Jackson ImmunoResearch Laboratories | 111-165-144 | 7.5 (IF) |
| Biotin conjugated Goat anti-mouse | Goat IgM | Jackson ImmunoResearch Laboratories | 115-065-020 | 2.6 (IF) |
| Biotin conjugated Goat anti-rabbit | Goat IgG | Jackson ImmunoResearch Laboratories | 111-065-144 | 2.8 (IF) |
| Cy2 conjugated streptavidin | - | Jackson ImmunoResearch Laboratories | 016-220-084 | 3.6 (IF) |
| Cy3 conjugated streptavidin | - | Jackson ImmunoResearch Laboratories | 016-160-084 | 3.6 (IF) |
| Streptavidin | - | Jackson ImmunoResearch Laboratories | 016-000-084 | 0.01 (IF) |
| Oregon Green 488 Phalloidin | - | Life Technologies | O7466 | 1.3 Units/ml |
| Peroxidase-AffiniPure Goat anti-rabbit | Goat IgG | Jackson ImmunoResearch Laboratories | 111-035-144 | 0.08 (WB) |
| Peroxidase-AffiniPure Goat anti-mouse | Goat IgG | Jackson ImmunoResearch Laboratories | 111-035-003 | 0.08 (WB) |

WB, IF and Co-IP signify western blotting, immunofluorescence and co-immunoprecipitation, respectively.
